# “The National Surgery Quality Improvement Project” (NSQIP): a new tool to increase patient safety and cost efficiency in a surgical intensive care unit

**DOI:** 10.1186/1754-9493-8-19

**Published:** 2014-04-24

**Authors:** John McNelis, Maria Castaldi

**Affiliations:** 1 North Bronx Health Network, Jacobi Medical Center, Bronx, NY, USA; 2 Department of Surgery, North Central Bronx Hospital, Rm 513, 1400 Pelham Parkway South, 10461 Bronx, NY, USA

**Keywords:** SICU, NSQIP, Pneumonia, Quality, Performance improvement

## Abstract

**Background:**

The “National Surgical Quality Improvement Program” (NSQIP) is a nationally validated, risk-adjusted database tracking surgical outcomes. NSQIP has been demonstrated to decrease complications, expenses, and mortality. In the study institution, a high rate of nosocomial pneumonia (PNEU) and prolonged ventilator days ≥48 hours (V48) was observed on the surgical service.

**Methods:**

The hospital studied is a 500 bed university-affiliated teaching hospital performing approximately 20,000 surgical operations per year. A multidisciplinary team was formed and a series of interventions were implemented to address high pneumonia rates and prolonged intubation. Specific interventions included enforcement of protocols and adherence to the Institute for Healthcare Improvement (IHI) ventilator bundles, including head of bed elevation, sedation holidays, extubate when ready, and early nutrition. NSQIP collected pre-operative through 30-day postoperative data prospectively on 1,081 surgical patients in the intensive care unit from January 1, 2010 – July 31, 2012. The variables pneumonia and V48 undergo logistic regression and risk adjusted results of observed versus expected are calculated. Mean and confidence intervals are represented in caterpillar charts and bar graphs. Statistical analysis was via Fisher exact t-test.

**Results:**

Progressive improvements were observed over a two-year period via three semiannual reports (SAR). Corrective measures showed a decrease in V48 with an observed to expected odds ratio (O: E) improving from 1.5 to 1.04, or 1.9% ( 7/368 patients) July 31, 2011 to 1.11% (12/1080 patients) July 31, 2012 respectively. Similarly, pneumonia rates decreased 1.36% (5/368 patients) July 31, 2011 to 1.2% ( 13/1081 patients) July 31, 2012 with O: E = 1.4 and 1.25 respectively. Statistical significance was achieved (p < .05).

**Conclusion:**

Given an estimated annual volume of 20,000 cases per year with a cost of $22,097 per episode of pneumonia and $27,654 per episode of prolonged intubation greater than 48 hours; a projected 32 avoided episodes of pneumonia and 160 avoided episodes of V48 could be realized with potential savings exceeding $5,000,000.

## Background

The “American College of Surgeons’ National Surgical Quality Improvement Program” (ACS NSQIP) database is a nationally validated risk adjusted program designed to measure surgical outcomes [1]. Since its inception in the Veterans Administration Hospital system in the 1980s, NSQIP has repeatedly been demonstrated to lead to improvements in surgical outcomes with increased savings. NSQIP has done so by decreasing complications and mortalities through benchmarked risk adjusted parameters [2]. Reductions of up to 27% in post-operative mortality and complication reductions of up to 45% have been described with incorporation of NSQIP into departmental Quality Assurance programs [3]. The availability of risk adjusted outcome data provides benchmarked targets to track the progress of quality initiatives [4]. In the study institution, post-operative pneumonia and prolonged ventilation greater than 48 hours were identified as problems in post-operative general surgery patients through both real time raw NSQIP data as well as the first benchmarked report. This was in keeping with Administrative data provided by the institution, as well as the clinical observations of the staff. As part of process improvement, data was analyzed, multidisciplinary teams were formed, corrective actions were taken and the outcome of the corrective action was monitored through NSQIP data. The authors hypothesized that access to NSQIP outcome data would result in a decrease in the ventilator associated complications observed at the study institution.

## Methods

The hospital studied is a suburban 500 bed University-Affiliated Teaching Hospital. It is an American College of Surgeons designated cancer center performing approximately 20,000 cases per year. The Hospital entered the NSQIP program in 2010, training two specialized Nurse Reviewers to collect data prospectively. The time frame examined spanned 2010–2012. As per NSQIP protocols, data acquired included demographics, peri-operative risk factors, laboratory data, operative variables as well as peri-operative and post-operative events. Specific outcomes measured in general surgical patients included mortality, cardiac events, nosocomial pneumonia, mechanical ventilation in excess of 48 hours, thrombo-embolic events, renal Failure, and surgical site Infection. Data was entered directly into a central NSQIP database and a comparative, risk adjusted SAR was generated detailing the hospital performance. The data was reported as both risk and non-risk adjusted being benchmarked against all NSQIP participating hospitals. In addition, real –time access to raw benchmarked (but not risk adjusted) data was available at any time. The risk-adjusted data was reported as a ratio of observed to expected incidence. The O: E calculation is made through a logistic regression to predict the probability of an outcome (i.e., expected outcome) of any event. The observed number of events is then divided by the expected number, yielding a risk-adjusted estimate of outcome. An O: E of 1.0 is defined as a hospital performance at the expected incidence of an observation. An O: E less than 1.0 would indicate that the observation occurs less than predicted while a ratio greater than 1.0 indicates a higher than anticipated incidence.

NSQIP uses precise and defined criteria to define each condition measured. Pneumonia is defined by both rigid radiographic and clinical parameters. Radiographically, the patient must demonstrate one chest radiological exam with a new or progressive and persistent infiltrate, a consolidation or opacity, or cavitation. Laboratory criteria to establish the diagnosis of pneumonia included the following: fever (>38.0 deg, C or >100.4 deg F.) with no other recognized cause, Leukopenia (<4000 WBC/mm3) or leukocytosis(≥12,000 WBC/mm3) and in addition, at least one of the following must be present: 5% Bronchoalveolar lavage (BAL) -obtained cells contain intracellular bacteria on direct microscopic exam (e.g., Gram stain), positive growth in blood culture not related to another source of infection, positive growth in culture of pleural fluid, positive quantitative culture from minimally contaminated lower respiratory tract (LRT) specimen (e.g. BAL or protected specimen brushing). Alternately at least two of the following: new onset of purulent sputum, or change in character of sputum, or increased respiratory secretions, or increased suctioning requirements, new onset or worsening cough, or dyspnea, or tachypnea, Rales or bronchial breath sounds, worsening gas exchange (e.g. Oxygen desaturations (e.g., PaO2/FiO2 ≤ 240), increased oxygen requirements, or increased ventilator demand) [5]. Only after these criteria are met will patients be diagnosed with Pneumonia.

After the establishment of baseline problem through the initial NSQIP report (SAR), a multidisciplinary team was assembled to examine the management of patients on mechanical ventilation. As a result, measures were implemented including enforcement of existing weaning protocols, extubation of patients when ready, early nutrition, early mobilization, strict adherence to the IHI ventilator bundles and frequent weaning trails in ventilated patients. Changes in the outcome data were tracked accordingly. Statistical analysis was via Fischer exact t-test.

## Results

**Figure 1:**
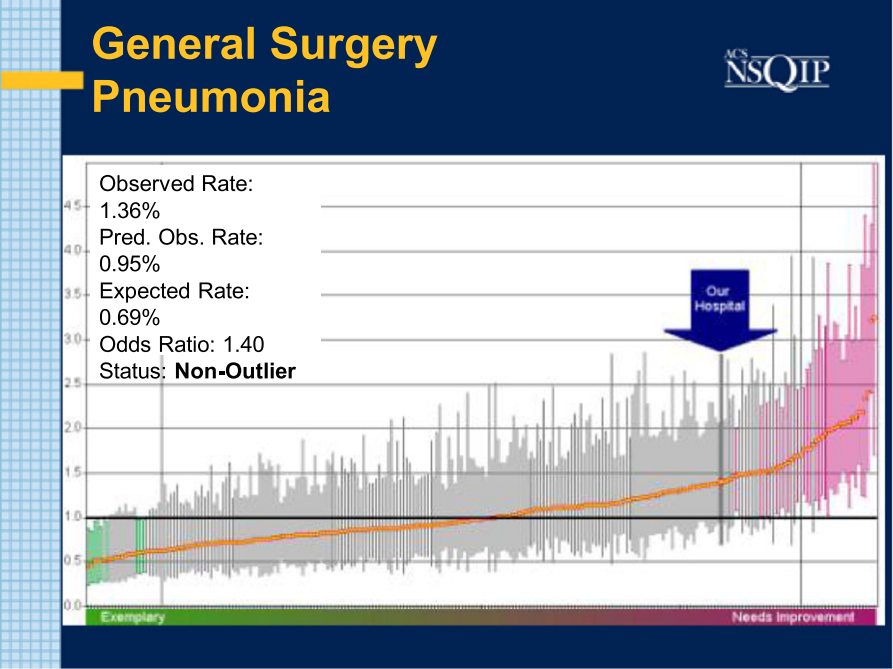
**SAR: PNEU July 2011.** Caterpillar chart with bold arrow noting study institution results for pneumonia. O: E odds ratio is 1.4. Each line corresponds to the result of one particular hospital. More successful performers lie to the left. Better than average hospitals show a Confidence Interval (CI) entirely below the mean (horizontal black line). Shaded green and pink are outliers.

**Figure 2:**
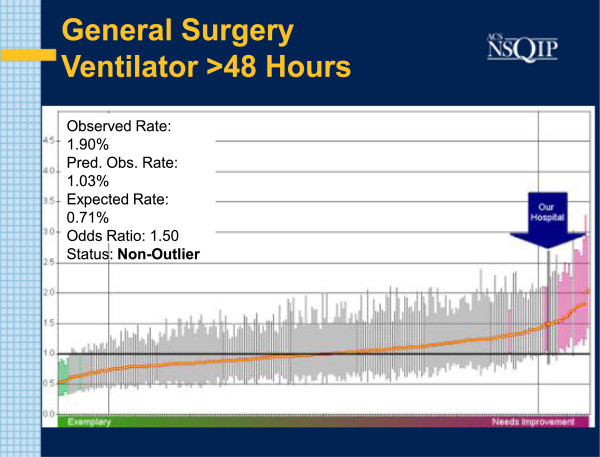
**SAR: V48 July 2011.** Caterpillar chart with bold arrow noting study institution results for prolonged ventilation. O: E = 1.5.

During the Study Interval, three Semi Annual Reports were generated: July 2011, January 2012 and July 2012. In the initial July 2011report, the O: E Odds Ratio for Pneumonia in General Surgery Patients was reported at 1.4. The absolute observed incidence was 5 cases in 368 cases examined( 1.36%). Associated with this was a higher than expected rate of patients remaining on the Ventilator for longer than 48 hours. The O: E incidence of patients on Ventilators for greater than 48 hours was 1.5. That constituted 7 occurrences in 368 patients examined (1.9%) (Figures 1 and 2).

**Figure 3:**
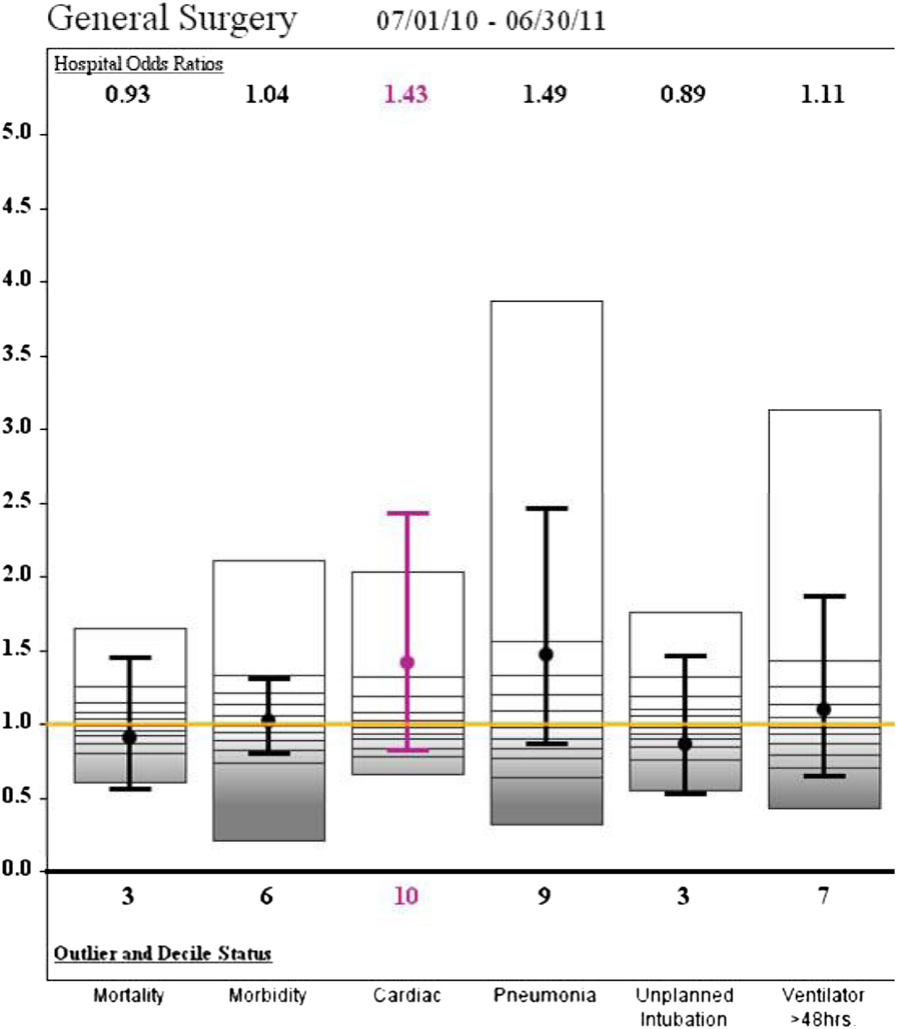
**SAR Jan. 2012.** Variables at study institution. Mean and confidence intervals included for each variable within the bar. The second SAR (Jan 2012) showing a decrease in prolonged ventilation. A drop in pneumonia was not initially observed.

**Figure 4:**
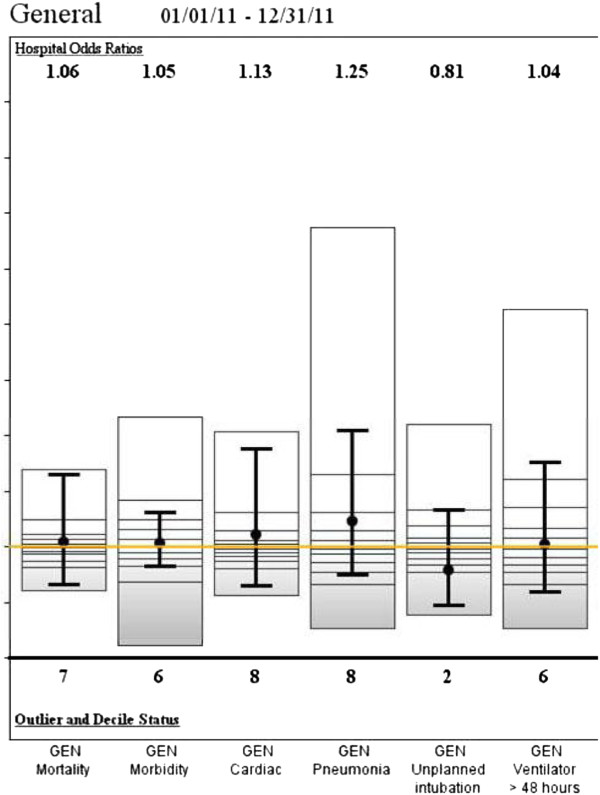
**SAR July 2012.** Variables at study institution. The third SAR showing a decrease in pneumonia rates.

By January 2012, the O: E for Pneumonia in General Surgery Patients had risen slightly to 1.48 (13/926, 1.4%) (NS), while the rate of O: E of patients on the Ventilator for greater than 48 hours declined to 1.11 (10/925, 1.08%) (Figure 3). In the final report of July 2012 the O: E for Pneumonia was 1.25 (13/1081, 1.2%) (p < .05) and the incidence of patients on the Ventilator less than 48 hours was 1.04 (12/1080, 1.11%) (p < 05) (Figure 4). The data is summarized in Table 1.

**Table 1:** Changes in Pneumonia and Prolonged Ventilation with associated savings

	**July 2011 SAR**	**Jan 2012 SAR**	**July 2012 SAR**	**Est. savings**
PNEU O:E (Actual%)	1.4 (1.36%)	1.48 (1.4%)	1.25 (1.2%)	$707,104
V48 O:E (Actual%)	1.5 (1.9%)	1.11 (1.08%)	1.04 (1.11%)	$4,424,640

## Discussion

Even before the initial NSQIP SAR, Pneumonia was identified as one of several areas of concern at the study institution. Indeed, the initial raw data provided indicated anticipated an increased Pneumonia incidence. In addition to the higher rate of pneumonia, the initial NSQIP SAR also reported an associated incidence of prolonged ventilation, as defined by intubation greater than 48 hours. Correlation between prolonged intubation and increased risk of pneumonia has been established, with the highest risk period being in the first two weeks post intubation [6]. In the study institution, the correlation was drawn between the increased incidence of pneumonia and prolonged intubation time. Initiatives to decrease the duration of Mechanical Ventilations would be followed by a subsequent improvement in pneumonia rates. In addition, processes in the care of ventilated patients were examined by a small multi-specialty Ventilator work group comprised of physicians, physician’s assistants, nurses and respiratory therapists. The group was subsequently expanded to include Physical Therapy and Nutritionists. Process measures, such as compliance with the IHI Ventilator bundles were re-examined. Compliance with IHI Ventilator bundles has been demonstrated to decrease the incidence of nosocomial pneumonia [7]. Review of the documented institutional compliance with the IHI Ventilator Bundles demonstrated a reported compliance rate of 100%. To validate this, a rapid experiment examining compliance with Head of Bed elevation, an easily quantifiable parameter, was undertaken. The findings demonstrated that while isolated measurements (snapshots) revealed 100% compliance at time of measurement, the average ventilated patients spent 59.9% of time with a head of bed elevation less than the recommended 30 degrees over a 24-hour period [8]. Consequently, all ventilator management processes were called into question. As a result, IHI Ventilator Bundle parameters were checked each shift with frequent re-evaluation during the 24-hour cycle. Special attention was given to maintenance of the Head of Bed above 30 degrees. In addition, examination of our extubation patterns, revealed a reluctance to wean and extubate in overnight hours- especially difficult post-operative patients. This policy of “resting” immediate post-operative patients in the Recovery Room often involved prolonged sedation and reliance on often unnecessary laboratory tests and radiographic imaging. To decrease ventilator time, a general policy was established to wean at all hours and extubate patients when ready. Prior to this, extubation practices were practitioner dependent, despite establishment of written guidelines. As such, the practice of sedating and not weaning patients overnight was ended and weaning continued over 24 hours. Variances in initiation of nutritional support were also observed and often found to be driven by practitioners outside the SICU team. As such, the critical care team undertook a policy of early enteral nutrition when possible. In addition, a program of early physical therapy and ambulation was also begun as early physical therapy and mobilization has been demonstrated to decrease the duration of mechanical ventilation [9].

The corrective measures implemented resulted in a decrease in prolonged ventilation by the second semi-annual report. Indeed, the SICU had made concerted efforts to begin earlier extubation in advance of the first report, which may account for the initial success. While a drop in pneumonia was not initially observed, the third NSQIP Semi-annual report showed a significant drop in the O: E ratio for Pneumonia in General Surgery from 1.4 to 1.25. This delay was most likely resultant from both a lag time in the effects of earlier weaning as well as an overlap in data reporting and implementation of corrective action. The O: E Odds Ratio for patients intubated for longer than 48 hours decreased from 1.5 to 1.04 as well. Interestingly, the enforcement and strict adherence to established protocols was also associated with a decreased incidence of unplanned intubations from an O: E of 1.05 to .81 (Figures 3 and 4). Hence the adherence to established guidelines seems to have resulted in earlier and safer weaning.

Financially, the decreases in pneumonia rate and ventilator days were associated with significant savings. Using the observed percentages, a decrease in pneumonia rate from 1.36% to 1.2% would translate into 32 less cases of pneumonia in a hospital with an average surgical volume of 20,000 cases per year. Similarly, a reduction on patients ventilated for longer than 48 hours from 1.9 to 1.11 would be associated with a net reduction of 160 episodes of prolonged intubation. Given an estimated cost of $22,097 per episode of Pneumonia and $27, 654 per episode of prolonged intubation beyond 48 hours, a net savings $707,104 annually for avoided pneumonia and $4,424,640 savings annually for decreased ventilator days [10,11]. These findings are consistent with the national NSQIP experience. Berunguer et al. reported a significant decrease in Surgical Site infections after initiating NSQIP [12]. Experience at Kaiser -Permanente in California achieved a Pneumonia rate of zero in surgical patients with and associated decrease in patients ventilated for greater than 48 hours [3].

## Conclusions

In the study interval, a progressive improvement in PNEU and V48 were observed. Process changes implemented in the SICU included enforcement and revision of existing weaning protocols, extubating patients when ready, early mobilization and nutrition. NSQIP data allowed for tracking and benchmarking of progress. Given an estimated annual Volume of 20,000 cases per year with a cost of $22,097 per episode of Pneumonia and $27,654 per Vent day greater than 48 hours, with 32 avoided episodes of Pneumonia and 160 avoided episodes of vent days greater than 48 hrs, potential savings of over five million dollars could be realized.

## Competing interests

The authors have no conflicts to declare.

## Authors’ contributions

JM- Study Design, First drafts, data collection, statistical analysis, MC- Manuscript re-write, statistical analysis, data collection. Both authors read and approved the final manuscript.
